# Electrical stimulation on adverse events caused by chemotherapy in patients with cervical cancer: A protocol for a systematic review of randomized controlled trial

**DOI:** 10.1097/MD.0000000000014609

**Published:** 2019-02-15

**Authors:** Peng-Hui Dou, Dan-Feng Zhang, Cui-Hong Su, Xiao-Li Zhang, Ying-Jie Wu

**Affiliations:** a Department of Radiotherapy and Chemotherapy; b Department of Gynecology; c Department of Scientific Research, First Affiliated Hospital of Jiamusi University, Jiamusi, China.

**Keywords:** adverse events, cervical cancer, chemotherapy, effectiveness, electrical stimulation, randomized controlled trial

## Abstract

**Background:**

This protocol of systematic review aims to investigate the effectiveness of electrical stimulation (ES) on adverse events (AEs) caused by chemotherapy in patients with cervical cancer (CC).

**Methods:**

This systematic review of randomized controlled trials will be identified through searchers of PUBMED, PsycINFO, Scopus, Opengrey, Cochrane Central Register of Controlled Trials, EMBASE, Cumulative Index to Nursing and Allied Health Literature, Web of Science, Allied and Complementary Medicine Database, and Chinese Biomedical Literature Database. All the sources will be searched from the inception to the date of study search ran. Additionally, websites of clinical trials registry and reference lists provided in relevant studies and reviews will also be searched. Two independent reviewers will evaluate the eligibility criteria of all potential literature, extract the data, and determine the risk of bias for each included study. RevMan 5.3 software will be used to pool the data and to conduct a meta-analysis.

**Results:**

This systematic review will assess the effectiveness of ES on AEs caused by chemotherapy in patients with CC.

**Conclusion:**

The findings of this study may summarize the latest evidence for the ES on AEs following chemotherapy for CC.

**PROSPERO registration number:**

PROSPERO CRD42019120191.

## 1 Introduction

Cervical cancer (CC) is one of the most common female malignancies and is also one of the leading causes of mortality in females worldwide.^[1–3]^ The previous study reported that there were 527,600 new CC cases and 265,700 deaths in females worldwide in 2012.^[4]^ In China, there were about 98,900 new CC cases in 2015. It accounted for 18.7% of the global incidence of CC among the female population.^[5]^ Several risk factors can result in CC, such as many sexual partners, early sexual activity, other sexually transmitted infections, a weak immune system, and smoking.^[6–8]^

Currently available managements mainly include chemotherapy, radiotherapy, and surgery,^[9–13]^ especially for chemotherapy. Thousands of clinical trials have reported that chemotherapy has achieved very satisfied efficacy.^[14–16]^ However, it also accompanies lots of severe adverse events (AEs), such as nausea and vomiting, fatigue, loss of appetite, pain, diarrhea, and so on.^[17–19]^ If these AEs cannot be treated fairly and timely, it may affect CC cure by reducing the dosage of chemotherapy, or even quit the chemotherapy. Thus, alternative interventions with fewer adverse reactions are urgently needed to treat those conditions caused by chemotherapy.

Fortunately, numerous clinical trials have reported that electrical stimulation (ES) can be used to treat AEs caused by chemotherapy effectively and safely.^[20–33]^ However, up to date, no systematic review has systematically investigated the effectiveness and safety of ES for AEs result from chemotherapy in patients with CC. Therefore, in this systematic review, we aim to assess the effectiveness and safety of ES for AEs caused by chemotherapy in patients with CC.

## 2 Methods and analysis

### 2.1 Study registration

The reports of this systematic review protocol have followed the Preferred Reporting Items for Systematic Reviews and Meta-Analysis Protocol statement guidelines.^[34]^ It has been registered on PROSPERO (CRD42019120191).

### 2.2 Eligibility criteria

#### 2.2.1 Types of studies

All randomized controlled trials (RCTs) comparing the effectiveness of ES on AEs caused by chemotherapy in patients with CC will be included, regardless the grades of AEs and the length of treatment period. Any other studies of non-RCTs, quasi-RCTs, case-control study, case reports, case series, review, comment, animal studies will all not be included.

#### 2.2.2 Types of participants

CC patients with any following AEs caused by chemotherapy will be included regardless of sex, age, and race. It includes fatigue, nausea and vomiting, pain, and diarrhea. However, patients will be excluded if they have all these disorders before the chemotherapy or result from any other conditions, except the chemotherapy.

#### 2.2.3 Types of interventions

In the experimental group, any types of ES, including electrical muscle stimulation, Russian ES, neuromuscular ES, functional ES, transcutaneous electrical nerve stimulation, and electroacupuncture regardless of dosage, treatment period will all be considered to include. If the study includes the ES plus other therapies, it will not be considered. Patients of the control group will be treated with any other interventions, but not any forms of ES.

#### 2.2.4 Types of outcomes

##### 2.2.4.1 Primary outcome

*Fatigue:* Fatigue severity, measured by The Multidimensional Fatigue Symptom Inventory-Short Form, Functional Assessment of Chronic Illness Therapy-Fatigue Scale, or the Brief Fatigue Index, or other relevant tools.

*Nausea or vomiting*: Frequency of nausea and vomiting.

*Pain*: Pain intensity, as measured by Visual Analog Scale, or Numeric Rating Scale, or any other relevant scales.

*Diarrhea*: Frequency of diarrhea, stool consistency.

##### 2.2.4.2 Secondary outcomes

Secondary outcomes include quality of life, as measured by the 36-Item Short Form Survey. In addition, adverse reactions are also evaluated.

### 2.3 Search methods for the identification of studies

#### 2.3.1 Search strategy

We will systematically retrieve the literature sources of PUBMED, PsycINFO, Scopus, Opengrey, Cochrane Central Register of Controlled Trials, EMBASE, Cumulative Index to Nursing and Allied Health Literature, Web of Science, Allied and Complementary Medicine Database, and Chinese Biomedical Literature Database from the inception to the date of study search ran. In addition, we will also retrieve the websites of clinical trials registry and reference lists provided in relevant studies and reviews. We have presented the search strategy sample for the Cochrane Central Register of Controlled Trials in Table 1. Any other databases and sources will also be retrieved by using the similar search strategy.

**Table 1:**
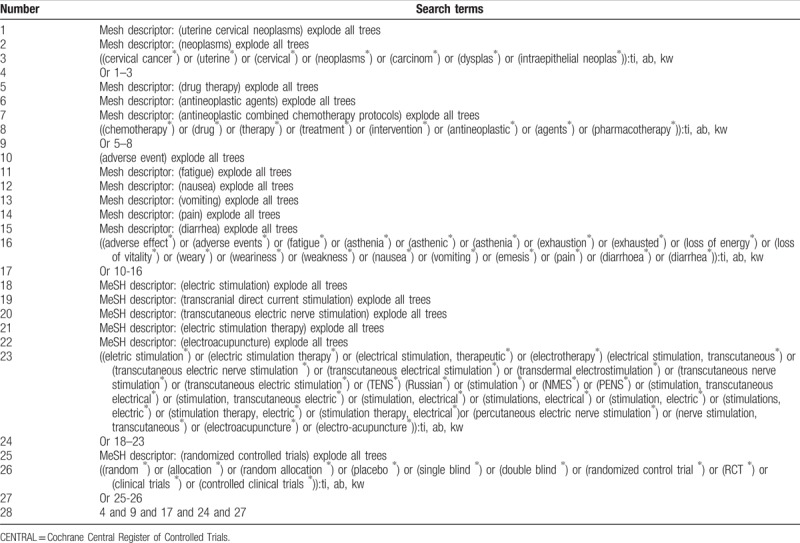
Search strategy applied in CENTRAL database.

#### 2.3.2 Study selection

**Figure 1:**
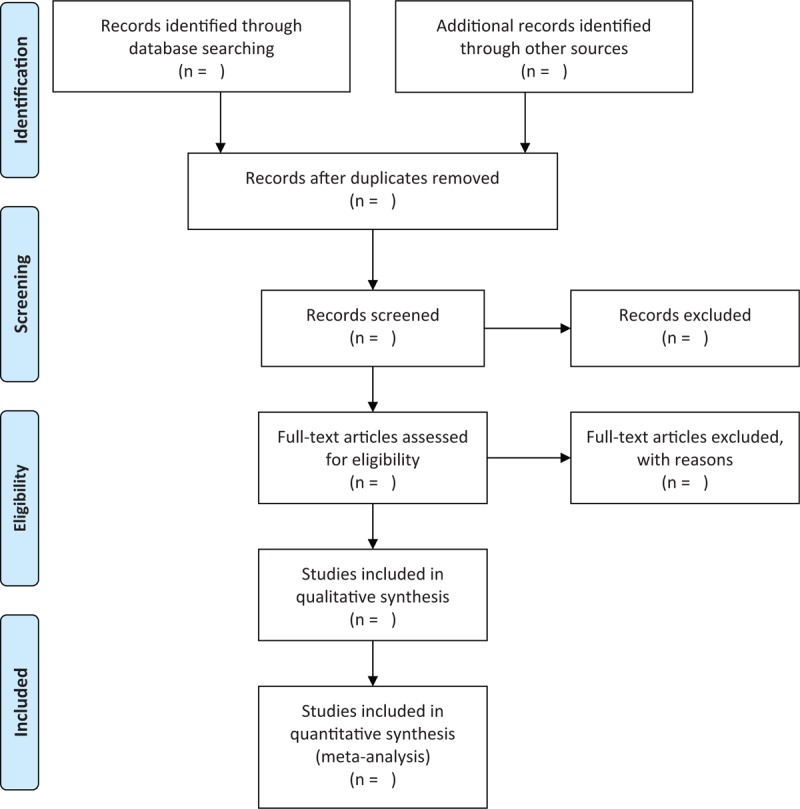
Flowchart of study selection.

Two reviewers will independently select the potential studies based on the predefined eligibility criteria. All the study selection process will be performed according to the PRISMA flowchart, and is presented in Figure 1. Any disagreements will be solved by consulting a third reviewer through discussion.

#### 2.3.3 Data collection

After selection, all the related data will be extracted from the included studies by using predefined data extraction forms. Two independent reviewers will carry out the data extraction. Any divergences of data extraction will be settled down by a third reviewer invited through discussion. The forms consist of the following information.

General information: title, first author, publication year, country, eligibility criteria, and patient characteristics.

Study methods: sample size, randomization, concealment, blinding, and any other potential risk of bias.

Intervention details: dosage, frequency, duration.

Outcomes: primary, secondary, and safety outcome measurements.

#### 2.3.4 Dealing with essential missing information

Any essential missing information, including missing data, will be inquired by contracting original authors to request. We will pool the available data only if the missing data cannot be achieved.

### 2.4 Risk of bias assessment

In this study, we will apply Cochrane risk of bias tool to assess the methodology quality for each included study. All the procedures will be performed by 2 independent reviewers. Disagreements between 2 reviewers will be resolved by a third review through discussion.

### 2.5 Data synthesis and analysis

RevMan 5.3 software will be used to pool and to analyze the data. Continuous data will be pooled and presented as mean difference with 95% confidence intervals (CIs). Dichotomous data will be pooled and presented as risk ratio with 95% CIs. Heterogeneity will be detected by using the Chi-square test and *I*^*2*^ values. The reasonable heterogeneity will be considered if *I*^*2*^ ≤50%, and pooled will be pooled by using a fixed-effect model. The significant heterogeneity will be considered if *I*^*2*^ >50%, and data will be pooled by using a random-effect model. Under such situation, subgroup analysis will be conducted. If there is still significant heterogeneity after the subgroup analysis, we will not pool the data, and carry out the meta-analysis. Instead, we will just report results as narrative description.

### 2.6 Additional analysis

#### 2.6.1 Subgroup analysis

Subgroup analysis will be carried out if the heterogeneity is substantial. It will be conducted according to different locations, study quality, treatment types, treatment duration, and outcome tools.

#### 2.6.2 Sensitivity analysis

Sensitivity analysis will be carried out to ensure the robustness and stability of pooled results data by removing low-quality trials.

#### 2.6.3 Reporting bias

If sufficient eligible studies are included, the potential reporting bias will be identified by funnel plots.^[35]^ Additionally, Egg regression test will also be performed to check the asymmetry of funnel plots.^[36]^

## 3 Discussion

To our best knowledge, although lots of clinical trials regarding the effectiveness of ES on AEs caused by chemotherapy in patients with CC were conducted,^[20–33]^ no systematic review specifically focused on the ES for AEs caused by chemotherapy in patients with CC. Therefore, the purpose of this study is to evaluate the effectiveness of ES on different AEs resulted from chemotherapy on CC. The results of this study will provide most present evidence on the effectiveness of ES for the treatment of AEs caused by chemotherapy in patients with CC. Its findings may also provide helpful evidence for the clinical practice, and researchers for further study.

## Author contributions

**Conceptualization:** Peng-Hui Dou, Dan-Feng Zhang.

**Data curation:** Peng-Hui Dou, Dan-Feng Zhang, Cui-Hong Su, Xiao-Li Zhang, Ying-Jie Wu.

**Formal analysis:** Peng-Hui Dou.

**Funding acquisition:** Dan-Feng Zhang.

**Investigation:** Dan-Feng Zhang.

**Methodology:** Peng-Hui Dou, Cui-Hong Su, Xiao-Li Zhang.

**Project administration:** Dan-Feng Zhang.

**Resources:** Peng-Hui Dou, Cui-Hong Su, Xiao-Li Zhang, Ying-Jie Wu.

**Software:** Peng-Hui Dou, Cui-Hong Su, Xiao-Li Zhang, Ying-Jie Wu.

**Supervision:** Dan-Feng Zhang, Ying-Jie Wu.

**Validation:** Peng-Hui Dou, Dan-Feng Zhang, Xiao-Li Zhang, Ying-Jie Wu.

**Visualization:** Cui-Hong Su, Xiao-Li Zhang, Ying-Jie Wu.

**Writing – original draft:** Peng-Hui Dou, Dan-Feng Zhang, Cui-Hong Su, Ying-Jie Wu.

**Writing – review and editing:** Peng-Hui Dou, Dan-Feng Zhang, Cui-Hong Su, Xiao-Li Zhang, Ying-Jie Wu.
